# Successful Release of Penile Strangulation Caused by Rigid Constriction Device Using a Modified String Method: A Case Report

**DOI:** 10.1002/ccr3.73169

**Published:** 2026-07-16

**Authors:** Akinori Kato, Yozo Mitsui, Fumito Yamabe, Koichi Nakajima, Shota Otsuka, Hirotaka Sato

**Affiliations:** ^1^ Department of Urology Hokusuikai Memorial Hospital Mito Japan; ^2^ Department of Urology Toho University Omori Medical Center Tokyo Japan

**Keywords:** decompression, modified string method, penile strangulation, urological emergency

## Abstract

Penile strangulation is a rare urological emergency that may lead to ischemia or gangrene if not promptly treated. We report a case of a 52‐year‐old man with schizophrenia who presented 36 h after placing a metallic ring at the base of his penis for sexual stimulation. Physical examination revealed marked distal penile edema without evidence of necrosis. Initial attempts at manual removal were unsuccessful due to severe swelling. The device was subsequently removed safely using a modified string method combined with needle puncture for decompression. This approach enabled reduction in penile volume and facilitated successful removal without complications or the need for surgical intervention. The modified string method represents a minimally invasive, effective, and safe technique for the management of rigid constriction devices, particularly in cases of low‐grade penile strangulation.

**Key Clinical Message**

The modified string method combined with cavernous aspiration provides a simple and minimally invasive approach for penile strangulation. Decompression of edematous tissue facilitates safe and efficient removal of constricting devices, even in prolonged cases.

## 1 Introduction

Penile strangulation is a rare urological emergency condition commonly caused by the placement of constricting devices such as rings around the penis, often for an autoerotic purpose [1]. Delayed treatment can lead to progressive edema, tissue necrosis, and/or urinary retention, possibly resulting in penile amputation in severe cases [2]. Prompt removal of the constriction device is essential, with the chosen removal technique dependent on the severity of strangulation and the object used.

When penile constriction devices are made of metal, removal frequently requires technically demanding or more invasive procedures. The string method is a well‐known, simple, and minimally invasive technique commonly attempted first for the removal of constricting finger rings [3], while it has also been adapted for selected cases of penile strangulation [4, 5]. Noh et al. described a modified string technique that incorporates needle puncture for drainage [6]. This report presents a case of penile strangulation caused by a rigid metal penile constriction device that was successfully treated using a modified string method, which enabled minimally invasive relief of compression.

## 2 Case History/Examination

A 52‐year‐old man with a history of schizophrenia was presented to the emergency department 36 h after placing a metallic ring at the base of his penis for sexual stimulation. Prior to arrival, the patient had unsuccessfully attempted self‐removal by making incisions on the glans penis surface.

## 3 Differential Diagnosis, Investigations, and Treatment

### 3.1 Differential Diagnosis

Based on the patient's presentation with penile swelling and a constricting metallic device, differential diagnoses included penile edema secondary to strangulation, ischemic penile injury, and early‐stage penile necrosis. The absence of skin discoloration, sensory loss, and tissue breakdown suggested a low‐grade injury without irreversible ischemic damage [7, 8].

### 3.2 Investigations

A 52‐year‐old man with a history of schizophrenia presented to the emergency department 36 h after placing a metallic ring at the base of his penis for sexual stimulation. On examination, the patient reported mild penile pain (Numerical Rating Scale: 2) and difficulty in urination, although spontaneous voiding was preserved. Body temperature and blood pressure were within normal limits, and a stable general condition was noted.

**FIGURE 1:**
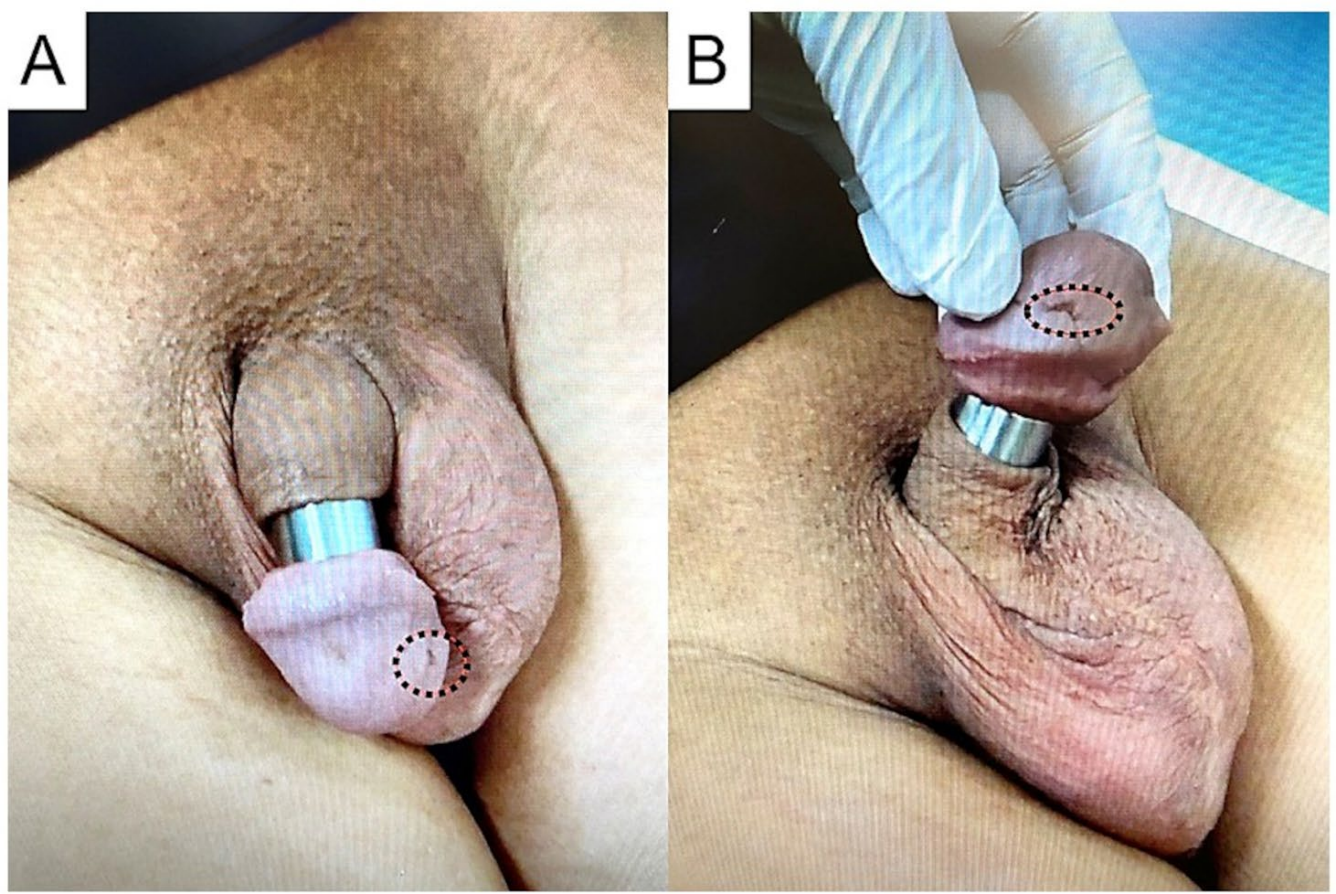
Clinical appearance at presentation. A rigid metal ring was positioned at the coronal sulcus, with edema observed distal to the ring. Multiple superficial lacerations, indicated by dotted black circles, were present on the glans penis (A: dorsal view; B: ventral view).

Physical examination revealed marked distal penile edema caused by a rigid metal ring positioned at the coronal sulcus. The ring measured approximately 1.5 cm in diameter, 1 cm in width, and 3 mm in thickness. Penile coloration and capillary refill were preserved, and no evidence of necrosis or loss of sensation was observed. Multiple superficial lacerations were present on the glans penis due to self‐inflicted attempts at removal (Figure 1A, dorsal view; Figure 1B, ventral view). Based on these findings, the injury was classified as Grade I (low‐grade penile injury) (Table 1) [7, 8].

**TABLE 1:** Classification of penile strangulation injury severity.

Bhat	Silberstein
Classification system [7]	Classification system [8]
Grade 1	
Distal penile edema without evidence of skin ulceration or urethral injury	
Grade 2	
Skin injury and constriction of the corpus spongiosum without urethral injury; distal penile edema with decreased sensation	**Low grade**
Grade 3	
Injury to the skin and urethra without urethral fistula formation; loss of distal penile sensation	
Grade 4	
Complete division of the corpus spongiosum resulting in urethral fistula, with constriction of the corpus cavernosum and loss of distal penile sensation	**High grade**
Grade 5	
Gangrene, necrosis, or complete penile amputation	

### 3.3 Treatment

Initial attempts at manual removal of the constriction device were unsuccessful due to severe distal edema and limited space between the penile tissue and the ring.

A modified string method was subsequently performed as a minimally invasive approach. A 45‐cm 4‐0 nylon suture was selected because it was sufficiently strong to withstand traction while remaining thin and flexible enough to pass beneath the metal constriction device. The suture was carefully passed between the dorsal penile surface and the metallic ring using mosquito forceps, and the proximal end was secured with forceps. An 18‐gauge needle was then inserted into the glans penis, and blood was aspirated from the corpus cavernosum to achieve decompression and reduce penile volume.

**FIGURE 2:**
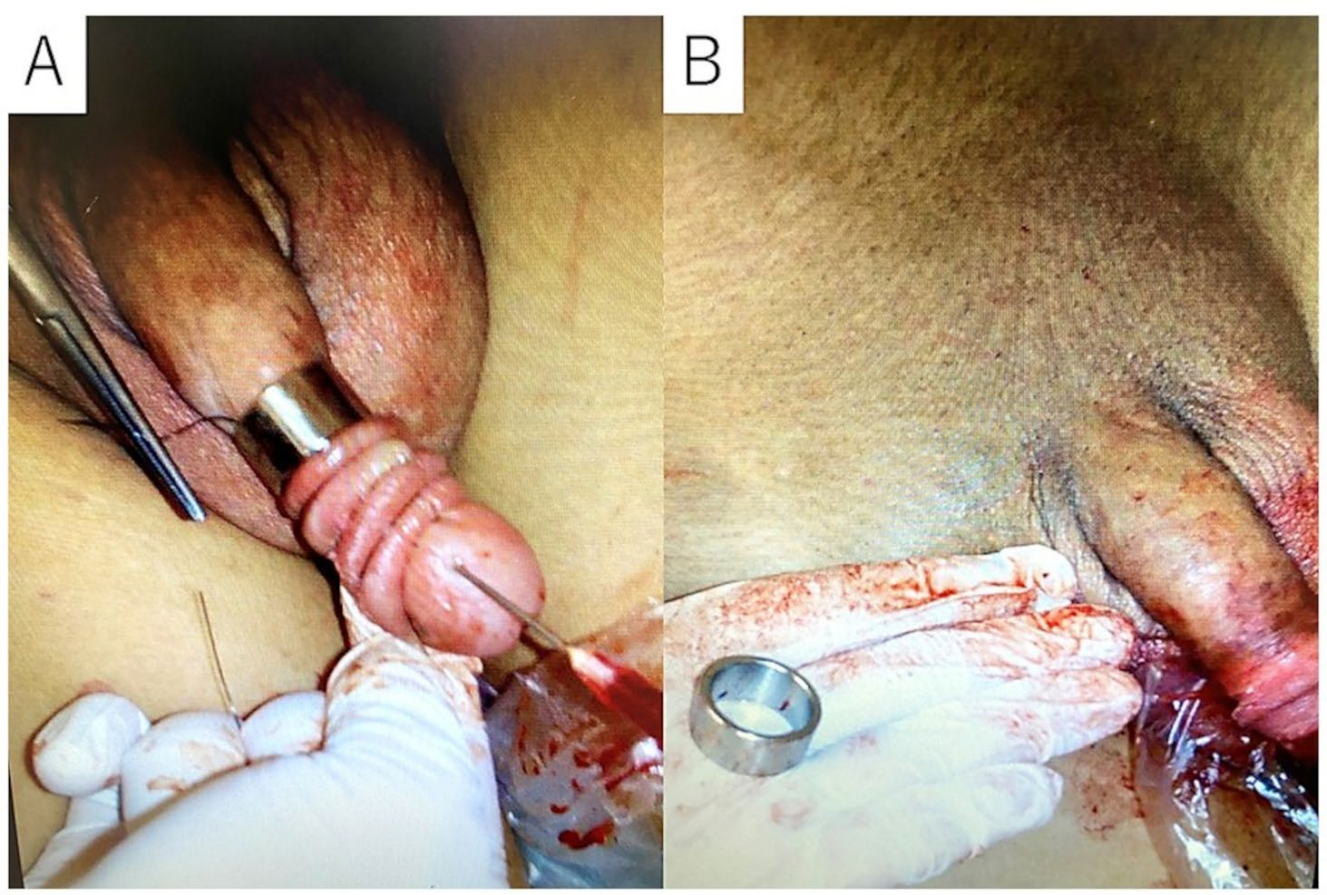
Device removal using the modified string method. (A) A suture was wrapped several times around the distal part of the penis to achieve circumferential compression and facilitate continuous blood drainage through an 18‐gauge needle. (B) Following reduction in penile edema, the metallic ring was gradually guided toward the glans penis and subsequently removed.

**FIGURE 3:**
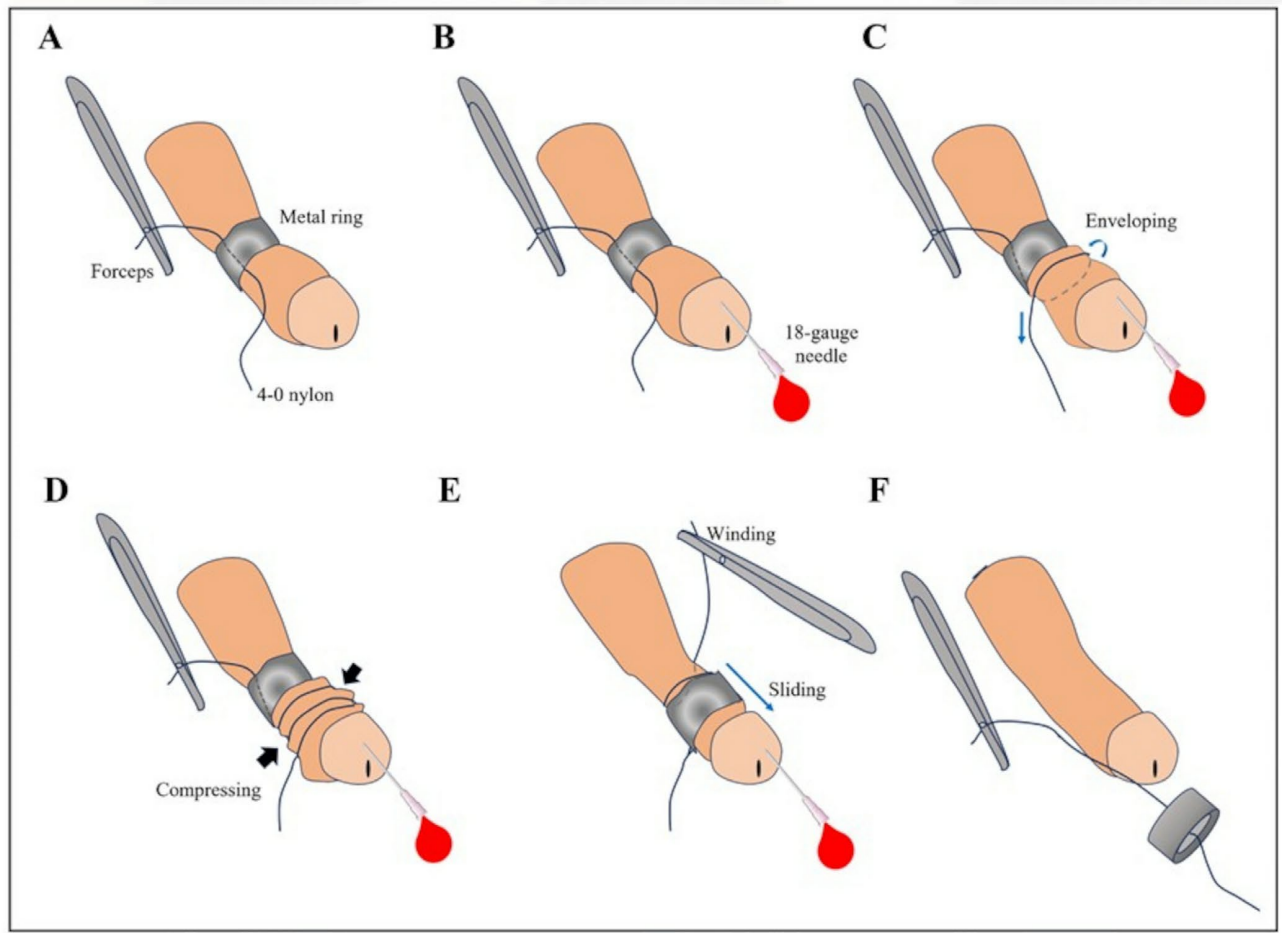
Stepwise procedure of the modified string method. (A) A nylon suture was passed between the dorsal surface of the penis and the metallic ring, and the proximal end of the suture was secured with forceps. (B) An 18‐gauge needle was inserted into the glans penis, then blood was aspirated from the corpus cavernosum to achieve decompression. (C) The distal portion of the suture was wrapped around the distal penis. (D) The suture was wrapped repeatedly to compress the edematous tissue and provide further blood drainage. (E) As the proximal suture was gradually unwound, the metallic ring was advanced distally. (F) The metallic ring was ultimately removed together with the suture.

The distal portion of the thread was wrapped circumferentially around the edematous penis to provide compression, while continuous drainage through the needle was maintained to enhance decompression (Figure 2A). As the proximal thread was gradually unwound, the constricting ring was advanced distally and successfully removed (Figure 2B). The procedure was completed without anesthesia and without complications [6]. Detailed illustrations of the procedure used for the modified string method are provided in Figure 3. Following several days of hospitalization, the patient was discharged without needing intervention and had not experienced subsequent complications at the time of writing.

## 4 Conclusion

A rare case of penile strangulation caused by a rigid metallic ring was successfully treated using the modified string method. This method represents a simple, safe, and effective minimally invasive approach for low‐grade penile strangulation injury and may be considered as a first‐line treatment option before proceeding to surgical intervention.

## 5 Discussion

Penile strangulation caused by various constricting objects represents a type of compartment syndrome that can progress to penile ischemia and infarction. Urgent intervention is required, as delayed treatment can result in serious complications, including sepsis and penile amputation. Notably, the severity of such penile injuries can significantly increase when treatment is delayed beyond 72 h [8]. Prompt removal of the device causing penile strangulation, aimed to achieve rapid decompression and restore blood flow, is the primary treatment. Nonmetallic devices, such as threads and rubber bands, can exert high pressure on the penis due to their elasticity, leading to progressive pressure increase and tissue injury worsening, although they are generally easy to remove [8]. In contrast, while metallic devices may initially cause less damage, the methods used for their removal are often more invasive and difficult [8]. Techniques for the removal of metallic constriction devices, presented in order of least to most invasive, include the string method with or without penile aspiration, use of a non‐powered cutting instrument, and use of an electrically powered cutting device. Selection of appropriate treatment for each case is crucial for timely and safe removal of metallic devices within a limited therapeutic window.

The Bhat classification system classifies penile strangulation into five categories [7]. Silberstein et al. simplified this classification into two groups: low grade (Types I–III) and high grade (Types IV and V), with the latter likely requiring surgical intervention [8]. A systematic review of 100 cases by Campbell et al. found that 36 were caused by metal rings, of which four were successfully treated with the string technique [9]. Notably, three of those four cases (75%) were classified as low grade and achieved favorable outcomes with conservative treatment. Accordingly, in cases of low‐grade penile strangulation injuries, initial removal of metallic devices using a minimally invasive technique such as the string method is recommended [8]. In the present case, despite a relatively prolonged strangulation time of 36 h, the injury remained low grade (Grade I), characterized by distal edema without tissue necrosis or sensory loss. One possible explanation for this limited severity is the mechanical nature of the constricting device. Unlike elastic materials, rigid metallic rings do not exert progressively increasing pressure, which may allow partial preservation of arterial inflow despite venous outflow obstruction. This imbalance can result in edema without complete ischemia. Additionally, the relatively small size of the ring may have allowed residual microcirculation, preventing progression to a high‐grade injury. Based on these considerations, we propose a practical treatment strategy. For low‐grade penile strangulation caused by metallic rings, a minimally invasive approach such as the modified string method should be considered as first‐line treatment. When severe edema limits manual removal, adjunctive decompression techniques, including corporal aspiration, can enhance its effectiveness. In contrast, high‐grade injuries or failed minimally invasive attempts should prompt early transition to cutting devices or surgical intervention.

The modified string method used in this case combines circumferential compression with corporal aspiration to achieve rapid reduction in penile volume. Continuous aspiration during compression may further enhance decompression and facilitate device removal. This approach offers advantages including reduced risk of iatrogenic injury, avoidance of specialized equipment, and feasibility at the bedside, although it may be less effective in severe or high‐grade cases.

In conclusion, this case underscores the importance of individualized management strategies in penile strangulation. The modified string method with adjunctive aspiration is an effective and minimally invasive option for selected low‐grade cases. Furthermore, injury severity may be influenced not only by the duration of strangulation but also by the mechanical properties of the constricting device, which should be considered in clinical decision‐making.

## Author Contributions

**Akinori Kato:** conceptualization, project administration, writing – original draft, data curation. **Shota Otsuka:** methodology. **Koichi Nakajima:** supervision. **Yozo Mitsui:** conceptualization, project administration, writing – review and editing. **Hirotaka Sato:** supervision. **Fumito Yamabe:** data curation.

## Funding

The authors have nothing to report.

## Ethics Statement

The authors have nothing to report.

## Consent

Written informed consent was obtained from the patient for publication of this case report and accompanying images.

## Conflicts of Interest

The authors declare no conflicts of interest.

## Data Availability

Data sharing not applicable to this article as no datasets were generated or analysed during the current study.
